# Effect of Catechin on the Formation of Polycyclic Aromatic Hydrocarbons in *Camellia oleifera* Oil during Thermal Processing

**DOI:** 10.3390/foods12050980

**Published:** 2023-02-25

**Authors:** Wenjun Pei, Jiaqi Wang, Lu Zhang, Yiwen Guo, Minjie Cao, Ruijie Liu, Ming Chang, Xingguo Wang

**Affiliations:** 1 National Key Laboratory, School of Food Science and Technology, Jiangnan University, 1800 Lihu Road, Wuxi 214122, China; 2 China National Center for Food Safety Risk Assessment, Southeast of the Intersection of Huangmuchang Road and Dongbai Street, Chaoyang, Beijing 100022, China

**Keywords:** *C. oleifera* oil, polycyclic aromatic hydrocarbon, phenolic compounds, lipid oxidation

## Abstract

Polycyclic aromatic hydrocarbons (PAHs) in oil are affected by many factors, including temperature, time, and PAHs precursors. Phenolic compounds, as beneficial endogenous components of oil, are often associated with the inhibition of PAHs. However, studies have found that the presence of phenols may lead to increased levels of PAHs. Therefore, this study took *Camellia oleifera* (*C. oleifera*) oil as the research object, in order to study the effect of catechin in the formation of PAHs under different heating conditions. The results showed that PAH4 were generated rapidly during the lipid oxidation induction period. When the addition of catechin was >0.02%, more free radicals were quenched than generated, thus inhibiting the generation of PAH4. ESR, FT-IR, and other technologies were employed to prove that when the catechin addition was <0.02%, more free radicals were produced than quenched, causing lipid damage and increasing PAHs intermediates. Moreover, the catechin itself would break and polymerize to form aromatic ring compounds, ultimately leading to the conclusion that phenolic compounds in oil may be involved in the formation of PAHs. This provides suggestions for the flexible processing of phenol-rich oil to balance the retention of beneficial substances, and for the safe control of hazardous substances in real-life applications.

## 1. Introduction

PAHs are a class of organic compounds that contain multiple (≥2) aromatic rings commonly found in various natural or artificial media we are exposed to daily, such as water, soil, air, and food [1]. Several studies have shown that long-term exposure to PAHs can lead to many adverse health outcomes, including abnormal lung function, chronic obstructive pulmonary disease, and various cancers [2]. As a result of their lipophilic and hydrophobic properties, PAHs are more likely to accumulate in the food chain [3], and are usually produced during high-temperature processes, such as grilling, frying, smoking, baking, etc. [4]. Among them, oil is the primary source of dietary exposure to PAHs. Even so, the exact mechanism of PAHs formation in oil has not been well verified; however, some researchers believe that PAHs formation is strongly related to fats, and speculate that fatty acids generate many free radicals during their oxidation to hydroperoxides, which form PAHs through reactions such as intramolecular addition or small molecule polymerization [5]. Oil is an essential part of the human diet, and *C. oleifera* oil is a vegetable oil rich in beneficial ingredients and is widely used by consumers. Considering the susceptibility of edible oil to PAHs and their risk to human health during daily high-temperature use, it is crucial to investigate the causes of PAHs generation in *C. oleifera* oil, in order to control PAHs contamination. 

Previously, numerous studies have shown that phenolic compounds can inhibit the formation of PAHs due to their antioxidant properties [6]; however, it was found that the contents of phenolic compounds, phytosterols, and another lipid companions in *C. oleifera* oil extracted by the hot pressing process increased with an increase in temperature, while the contents of Benzo(a)pyrene (BaP) also increased [7]. There are also studies found where the contents of PAH8 [sum of benzo(a)anthracene, chrysene, benzo(b)fluoranthene, benzo(k)fluoranthene, benzo(a)pyrene, dibenzo(a,h)anthracene, benzo(g,h,i)perylene, and indeno(1,2,3-c,d)pyrene] in chargrilled chicken wings marinated with black tea high in phenolic contents were much higher than that in control groups [8]. Min et al., as well as others, found higher levels of PAH8 in their heated meat model system than in the control group when EGCG [(−)-epigallocatechin gallate] was used as an antioxidant at concentrations of 50 μg/kg and 100 μg/kg [9]. We also found in this study that the rate of increase of PAHs in crude camellia oil (CCO) was faster than that in refined camellia oil (RCO) under the same heat treatment conditions. Therefore, we speculated whether some substances in the CCO caused an increase in PAHs after heat treatment. Based on this, we found that many studies pointed out the presence of the precursors of PAHs in food, such as amino acids, lipids, polyphenols, cellulose, etc. These precursors may undergo condensation polymerization to form PAHs under high-temperature conditions [10,11]. 

Therefore, the aim of this study was to elucidate the effect of phenols present in *C. oleifera* oil, which may be PAHs precursors, on the formation of PAHs and the mechanism of their formation. In order to detect accurately the PAHs formed after heating, changes in PAH4 [sum of benzo(a)anthracene, benzo(b)fluoranthene, chrysene, benzo(a)pyrene] were monitored in this study, which was stated to be a more suitable approach to quantify the contamination level of PAHs in oil [12]. The findings of this study will provide valuable and innovative reference data, as well as scientific basis for the control of PAHs in edible oils.

## 2. Materials and Methods

### 2.1. Materials

The CCO and RCO were obtained from Zhejiang Jiusheng Oil Seed Oil Co., Ltd. in Jiande City, Zhejiang Province. The standards of catechin were purchased from Sigma-Aldrich (St. Louis, MO, USA). Reagents for all experiments were obtained from Sinopharm Chemical Reagent Co., Ltd. (Shanghai, China).

### 2.2. Preparation of C. oleifera Oil with Different Catechin Contents and Heating Conditions

Preparation of oil samples containing different catechin concentrations: The catechin standard was accurately weighed, first dissolved in ethanol, then added corresponding volumes of ethanol solution to *C. oleifera* oil, and then dried under nitrogen at 35 °C. The final mass concentrations of catechin were 50, 100, 200, and 400 mg/kg, respectively, and stored at −4 °C. A blank group without catechin was set as the control. 

Preparation of heated oil samples for CCO and RCO: 500 mL crude oil and 500 mL refined oil were heated continuously at 180 ± 5 °C for 6 h in a thermostatic oil bath (Gongyi City Yuhua Instrument Co., Ltd., Zhengzhou, China). An aliquot of 60 mL oil was collected every 1 h during the 6 h continuous heating process and the collected samples were stored at −4 °C until further analysis.

Preparation of Rancimat pro-oxidation oil samples with catechin addition: The above catechin-added samples were heated with Rancimat to promote oxidation. The induction Period (IP) was measured with a Rancimat892 apparatus from Metrohm Nordic ApS (Glostrup, Denmark), at 140 °C, 160 °C, 180 °C, 200 °C, and 20 L/h airflows.

### 2.3. Analysis of PAHs

PAHs were detected by high performance liquid chromatography-fluorescence detection (HPLC-FLD) according to the previous study [13]. The correlation coefficient was greater than 0.999. The limits of detection (LOD) of Chr, BaA, BbF, and BaP in *C. oleifera* oil were 0.25, 0.29, 0.023, and 0.061 μg/kg. Meanwhile, the corresponding limits of quantitation (LOQ) were 0.85, 0.98, 0.076, and 0.21 μg/kg, respectively. The recovery rates of PAH4 ranged from 70.2% to 110%.

Calculation of LOD and LOQ: An aliquot of 0.5 g oil sample was weighed and PAHs mixed standard solution was added quantitatively to achieve concentrations of 2 μg/kg, 10 μg/kg, and 20 μg/kg, respectively, for 6 repetitions. The detection limit of the object to be measured was set according to international regulations [14]. The concentration under S/N = 3 is defined as LOD, while the concentration under S/N = 10 is defined as the limits of quantitation LOQ.

### 2.4. Determination of Physicochemical Parameters, Fatty Acid Composition, Minor Constituents Content, and Heating Products 

The physicochemical parameters (acid value and peroxide value) of this study were determined according to the AOCS official method [15]. Fatty acid composition was analyzed by Gas Chromatography (GC) according to the method reported previously [16]. Minor constituents contents (tocopherols, phytosterols, and total phenols) and PAHs intermediates were analyzed according to the previous study [17], and some modifications were made on this basis. Details are provided in the Supplementary Materials to the manuscript.

### 2.5. Electron Spin Resonance (ESR) Spectroscopy

ESR spectra were measured using a Bruker spectrometer (BrukerGmbH, Karlsruhe, Germany). Samples with and without catechin were used to detect free radical contents after heating; accurately weighed 0.3% α-phenyl-n-tert-butyl nitrogen oxide (PBN), dissolved in the oil sample, mix thoroughly, and heat. Next, 100 µL of the heated oil sample was placed in a nuclear magnetic tube with an inner diameter of 4 mm. Then, it was placed in the resonant cavity of the measurement instrument. The ESR detection parameters refer to Chen [18].

### 2.6. Fourier Transform Infrared Spectroscopy (FT-IR) Analysis

The FT-IR spectrum of oil used a Fourier transform infrared spectrometer (Vertex 70, Bruker Banner Lane, Coventry, Germany). The main operating parameters of the instrument were as follows: measuring spectrum range of 4000~400 cm^−1^, scanning times of 64, resolution of 4 cm^−1^, and 2 times gain. Data were collected in log (1/R) format. After the collection program was programmed, the infrared spectrum of the sample was directly collected. Each sample was scanned three times, and its average spectrum was calculated and stored until analysis.

### 2.7. Identification of Catechin and Its Structures by High Performance Liquid Chromatography Ultraviolet Spectrometry (HPLC-UV) and Ultra Performance Liquid Chromatography Coupled with Quadrupole Time-of-Flight Tandem Mass Spetrometry (UPLC-Q-TOF/MS)

HPLC-UV was used to detect catechin. Briefly, the sample was dissolved with methanol, then the catechin in the sample were extracted by shock, followed by ultrasound for 20 min, and then placed in a −18 °C refrigerator for 10 min. After centrifugation, the supernatant was taken. Liquid phase and mass spectrometry detection conditions were referenced in previous studies [19,20].

### 2.8. Statistical Analysis

All experiments were performed in triplicate and results were expressed as means ± standard errors. Structures were produced with the use of ChemDraw. Statistical significance was analyzed by one-way ANOVA, using the SPSS 22.0 package (IBM, New York, NY, USA). The results were regarded as statistically significant when *p* < 0.05. Curve fits were performed using the Origin 8.0 (Electronic Arts Inc., Redwood City, CA, USA).

## 3. Results and Discussion

### 3.1. Changes in Physicochemical Properties, Lipid Companion, Fatty Acid Composition, and PAH4 of Crude C. oleifera Oil and Refined C. oleifera Oil during Heating

**Figure 1:**
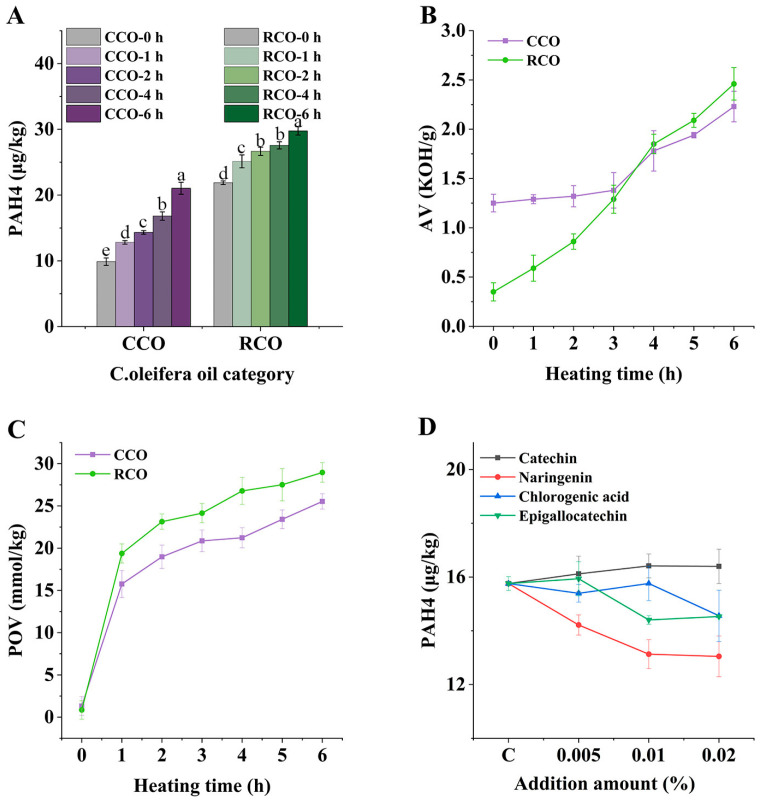
Changes of PAH4 in CCO and RCO heating for 6 h (**A**); The AV and POV changes of CCO and RCO during heating for 6 h (**B**,**C**); Effects of different addition of catechin, naringin, chlorogenic acid, and epigallocatechin on the formation of PAH4 in *C. oleifera* oil after heated at 180 °C (**D**). Bars with different letters show significant differences (*p* < 0.05).

Combined with previous studies, we found that the rate of increase of PAH4 in CCO and RCO differed under the same heating conditions. Figure 1A shows the change in PAH4 contents of CCO and RCO after heating at 180 °C for 6 h. It can be seen that the 21.88 μg/kg PAH4 contents in RCO are higher than that in CCO (9.88 μg/kg) without heating. The contents of PAH4 in both samples increased after heating, which may be due to the fact that high temperature is conducive to the formation of PAHs, through the massive cleavage of organic compounds [21]. After heating for 6 h, PAH4 in CCO and RCO both increased significantly (*p* < 0.05), results which were 82.61% and 36.01% higher than their initial PAH4 contents. It can be seen that the rate of increase of PAH4 in CCO was faster than that of RCO. This is a curious phenomenon, so this study performed a series of index measurements; thus, the main differences between CCO and RCO were identified and the reasons for this phenomenon were investigated.

The changes in fatty acids in *C. oleifera* oil under prolonged heating were firstly examined. As shown in Table 1, the main saturated fatty acids of CCO and RCO were palmitic acid and stearic acid, and the unsaturated fatty acids were oleic and linoleic acid. With the extension of heating time, the unsaturated fatty acids in CCO and RCO both decreased obviously, and palmitic acid contents gradually increased. 

Acid value (AV) and peroxide value (POV) are the most basic and important parameters to evaluate oil quality [22]. The changes in AV and POV of RCO and CCO during the heating process are shown in Figure 1B,C. They both underwent a continuous increase in AV and POV. It is clear, however, that the AV and POV growth rates in CCO are slower than those in RCO, which might be due to the fact that CCO contained more lipid companions.

Tocopherols, phytosterols, and polyphenols are natural nutrients in *C. oleifera* oil that affect the quality of the oil [23]. Changes in the contents of tocopherols, phytosterols, and total phenols during heating are shown in Table 1, from which it can be seen that the contents of tocopherols, phytosterols, and total phenols in unheated CCO were higher than those of the lipid companion in RCO. During the heating process, the contents of tocopherols, phytosterols, and total phenols in both oils underwent a significant reduction (*p* < 0.05). Tocopherols, phytosterols, and total phenols in CCO decreased by 82.5%, 22.5%, and 59.7%, respectively, and the RCO phytosterols and total phenols decreased by 10.7% and 42%, after 6 h heating, respectively. It can be seen that the loss of lipid companions in CCO was more serious throughout the heating process; as a result, it was speculated that some reactions of lipid companions play a role in the heating process of CCO, resulting in the differences between CCO and RCO.

In summary, it is worth exploring whether the growth of PAHs in CCO was the result of more abundant lipid companions. Through a series of experiments, combined with a literature review, we hypothesized that the increased production of PAHs in *C. oleifera* oil may be related to phenolic compounds. Therefore, we conducted correlation analysis of heating time, fatty acid composition, lipid companion, and PAH4 in *CCO* (Table 2), and found that the formation of PAH4 was negatively correlated with the reduction of total phenol, which suggested that the depleted phenolic compounds might be involved in the formation of PAH4. Therefore, we chose refined oil that had most of its lipid companions removed as the research object. We first studied the effect of phenolic compounds on the formation of PAHs in refined oil under high temperature, and then explored the mechanism of its influence on the formation of PAHs.

### 3.2. Effects of Catechin on the Formation of PAH4 in C. oleifera Oil under Different Heating Temperatures, Heating Times, and Catechin Additions 

According to previous research [8,9], several phenolic compounds present in *C. oleifera* oil were selected for separate addition experiments, and the change in PAH4 contents in the oil was examined (Figure 1D). This phenomenon made us think about why catechin caused an increase of PAHs in the heating process of oil. Therefore, we chose catechin as the research object, and explored its mechanism in the subsequent study.

**Figure 2:**
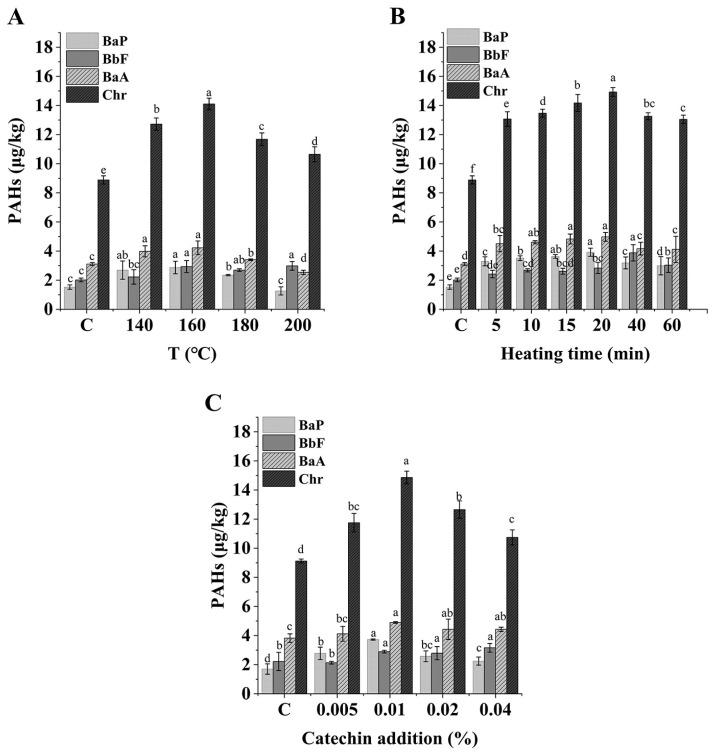
The PAH4 contents in *C. oleifera* oil at different heating temperatures under 0.01% catechin addition and 60 min heating time (**A**); The PAH4 contents in *C. oleifera* oil at different heating times under 0.01% catechin addition and 160 °C heated (**B**); and the PAH4 contents in *C. oleifera* oil at different catechin addition, heated at 160 °C for 60 min (**C**). Bars with different letters show significant differences (*p* < 0.05).

As shown in Figure 2A, at 0.01% catechin addition and 60 min heating time, the contents of BaP, BbF, BaA, and Chr increased with an increase in temperature. At 160 °C, they reached maximum values of 2.87, 2.94, 4.22, and 14.12 μg/kg, respectively, and then began to decrease. Therefore, 160°C was chosen as the experimental temperature for subsequent experiments. Additionally, the PAH4 contents showed the same trend with an extension in heating time when the catechin addition was 0.01% and the heating temperature was 160 °C (Figure 2B). After heating for 20 min, the PAH4 contents increased significantly compared with the control group (*p* < 0.01). Similar findings were found in the study of Li et al. [24].

In this study, the changes in PAH4 in *C. oleifera* oil during high-temperature oxidation were monitored under the rancimat pro-oxidation model, which is based on the principle of passing purified air into a sample that has reached a set heating temperature; the sudden increase in the conductivity of deionized water due to the release of volatile acids during oxidation is indicated as the end of the induction period, which can be defined as a measure of the antioxidant properties of the oil [25]. The IPs of *C. oleifera* oil used in this study were 110 min, 20 min, 10 min, and 6 min at 140 °C, 160 °C, 180 °C, and 200 °C, respectively. When the temperature of the system was higher than 160 °C, the contents of PAH4 increased first, and then decreased with an increase in temperature and time. This may have been due to the rapid decomposition and oxidation of oil in the induction period, leading to the rapid formation of PAHs in this system; meanwhile, in the later oxidation period, under the strong high-temperature reaction, PAHs may react with other components in the oil to produce other substances, resulting in a reduction in the PAHs already generated [26]. Therefore, the PAH4 in samples at 180 °C and 200 °C were lower than that at 160 °C when heated for 60 min. Furthermore, the PAH4 contents continued to increase in the first 20 min of the induction period when heated at 160 °C, and then during the period of 20–60 min, the oil was completely oxidized, and the contents of the PAH4 generated in the early stage gradually decreased under the severe reaction conditions, along with other components. Based on the above research, it could be concluded that temperature was an important factor in the formation of PAHs, which was also confirmed in a previous study [27]. As a result, the temperature was relatively lower at 140 °C compared to 160 °C, and the rate of increase of PAH4 contents was lower than that for PAH4 in the sample at 160 °C. Combined with the above experimental results, we chose 160 °C and 20 min as our experimental conditions.

Considering the results of both heating temperature and heating time, the formation of PAHs was influenced by both factors. Furthermore, the addition of catechin may have also had an effect on PAH4 production. For the purpose of finding the most effective concentration for PAH4 production, different additions of catechin (0.005%, 0.01%, 0.02%, 0.04%) were added. The samples were heated at 160 °C for 20 min, and the results are shown in Figure 2C. It was found that catechin had a significant effect (*p* < 0.005) on the increase in the formation of each PAH at additions of 0.005% and 0.01%. Most of the PAHs decreased slightly at a 0.02% concentration of catechin, with a significant decrease observed at a 0.04% concentration. Therefore, it was speculated that some cleavage and polymerization reactions of catechin at certain concentrations during the oxidation of oil at high temperatures may have led to an increase in PAH4 contents in oil.

The generation of PAHs in oil at high temperatures is mainly due to the cleavage and oxidation of triglycerides in oil, resulting in the production of cyclized alkane compounds; these, in turn, lead to the generation of a large number of aldehydes, ketones, and hydrocarbons; at the same time, this generates a large number of free radicals, eventually leading to a significant increase in PAHs [28]. However, it is very difficult to study the formation process of PAHs via intermediates because some intermediates formed by PAHs are very active and the process is transient. Only some relatively stable intermediates can be used to speculate on the formation process of PAHs. Therefore, in order to investigate the main mechanism of the increase in PAHs contents in *C. oleifera* oil caused by catechin, the roles of catechin addition on fatty acid composition, PAHs intermediates, free radical intensity, and quantity of *C. oleifera* oil at high temperature were further determined.

### 3.3. Effect of Catechin on Fatty Acid Composition during Heat Treatment

In order to explore the effect of catechin on the fatty acid stability of *C. oleifera* oil under high-temperature conditions, the changes in fatty acid composition under different conditions of catechin addition were studied. As shown in Table 3, after heating at different temperatures for 60 min, the contents of oleic acid and linoleic acid decreased significantly with an increase in temperature, and the contents of linoleic acid decreased. As the heating time increased from 0 to 60 min, samples with 0.01 % catechin at 160 °C showed a significant increase in palmitic and stearic acids from the 20th min and a significant decrease in oleic and linoleic acids from the 15th min. This result was in keeping with the results of Bansal et al. [29]. Under high temperature, unsaturated fatty acids in *C. oleifera* oil are very unstable and highly susceptible to oxidation and degradation; thus, some unsaturated fatty acids will generate saturated fatty acids and lead to an increase in palmitic and stearic acid contents. When the samples without catechin were compared with those containing catechin, significant changes in fatty acids began to occur after heating at 160 °C for 20 min (*p* < 0.05). When catechin was added at 0.02% and 0.04%, the contents of oleic acid and linoleic acid were higher than those of the control group, which indicated that catechin could inhibit the degradation of unsaturated fatty acids at these concentrations. When catechin was added at 0.01%, the contents of oleic acid were significantly lower than those of the control group, and the contents of linoleic acid were lower than that of the control group when catechin was added at 0.005 %. These results were similar to those obtained by Yuan [30]. This indicated that catechin may affect fatty acid decomposition and oxidation after heat decomposition when the addition amount of catechin was 0.005 or 0.01%.

### 3.4. Effect of Catechin on PAHs Intermediates during Heating

The research found that the degradation of oil in the process of the high-temperature thermal reaction itself produced free radicals, oleic acid, and other unsaturated fatty acids that are oxidized to generate hydroperoxides. Hydroperoxides are extremely unstable, and will be further cleaved to produce small molecules of substances, generating hydroxyl radicals and alkoxy radicals, alkoxy radicals further break down, thereby generating aldehydes, olefins, and other compounds [31]. Aldehydes are important substrates for some hazard factors such as heterocyclic amines, polycyclic aromatic hydrocarbons, etc. Liu Li found that when aldehydes were added to oil alone, a significant increase in PAHs occurred in the system [32]. Studies also have shown that the addition of nutrients, such as tea polyphenols to oil, can intensify the production of aldehydes in oil at high temperatures [33]. Therefore, it was presumed that the addition of catechin to *C. oleifera* oil could promote the production of aldehydes and eventually lead to an increase in PAH4 contents.

**Figure 3:**
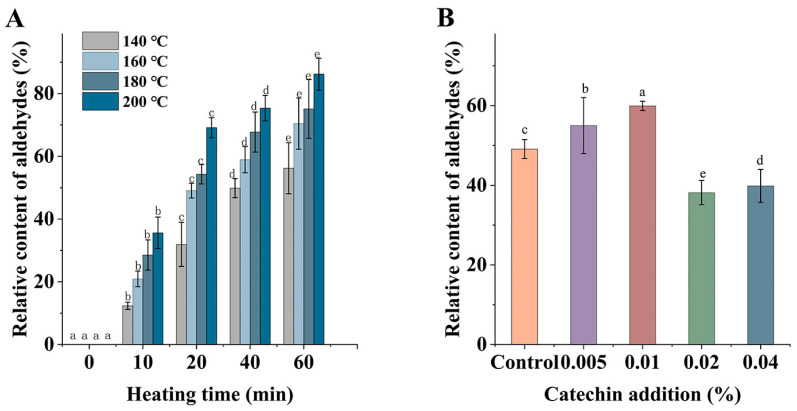
Relative contents of aldehydes in *C. oleifera* oil under different heating temperatures and times (**A**); Relative contents of aldehydes after adding different catechin addition and heated at 160 °C for 20 min (**B**). Bars with different letters show significant differences (*p* < 0.05).

Figure 3A displays the graph of the relative content changes of aldehydes (RCA) generated in *C. oleifera* oil after heating at different temperatures (140 °C, 160 °C, 180 °C, and 200 °C) for 60 min. A total of 13 aldehydes were detected in this experiment, including nonanal, octanal, 2-undecenal, etc. It can be seen that high-temperature treatment led to a large number of aldehydes generated, and the overall contents levels also differed; the RCA continued to increase with increasing temperature during the first 20 min induction period of oil oxidation, from 0% to 31.92, 49.1, 54.31, and 69.12%, and the increasing trend was similar to that of PAH4. When in the oxidation period of oil, the primary oxidation products generated in the induction period were rapidly degraded to generate secondary oxidation products, and volatile compounds such as aldehydes were produced in large quantities in this phase, so that RCA continued to increase; however, the PAHs generated in the induction phase were rapidly depleted in this phase, and the rate of PAHs consumption in this phase was faster than the rate of PAHs production from aldehydes. Thus, this may have led to a continuous increase in aldehydes, but a decrease in PAH4. Figure 3B shows the changes in RCA produced by the heat treatment of *C. oleifera* oil with different additions of catechin. It can be seen that the RCA were higher in the samples with 0.005% and 0.01% catechin (59.94% and 55.01%), which were significantly higher than that of the control group (*p* < 0.05). This was probably attributed to the addition of 0.005% and 0.01% catechin that caused the oxidative degradation of fatty acids, which led to an increase in RCA. When the samples with 0.02% and 0.04% of catechin were added, the RCA were 38.17% and 39.86%, respectively, which were lower than the control group; this may have been due to the fact that the high concentration of catechin captured the aldehydes in the system, and thus reduced the contents of aldehydes in the samples. 

### 3.5. Electron Spin Resonance Studies on the Mechanism of PAH Formation

ESR is a modern analytical method for the direct and effective detection of compounds containing unpaired electrons [34]. The free radicals generated during the oxidation of oil can be trapped, which is also particularly valuable for studying the mechanism of free radical generation with PAHs.

**Figure 4:**
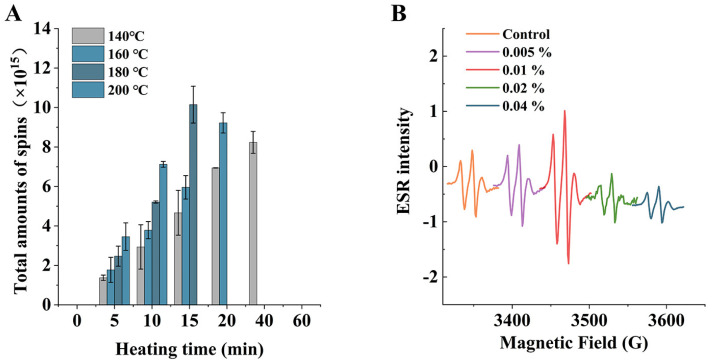
Total amounts of spin under different heating temperatures and times (**A**); ESR spectrum of different catechin addition at 160 °C for 20 min (**B**).

Figure 4A shows the total number of free radicals at different temperatures (140, 160, 180, and 200 °C) with different heating times. It can be seen from the figure that the higher the temperature, the higher the total number of spins at the same heating time; the total number of radicals increased to 2.94, 3.79, 5.21, and 7.12, respectively, at 10 min, and the number of radicals also showed a trend of increasing and then decreasing with time. Based on the above results, it can be seen that during the induction period of oil oxidation, the free radicals were more active and the total number of spins increased, while no free radicals were detected after heating at 160 °C, 180 °C, and 200 °C for about 20 min. This may have been due to the fast bimolecular reaction rate between the newly generated lipoxy radicals and radical adducts, leading to rapid quenching of radical adducts in the system. According to the preliminary literature review and experiments, we hypothesized that the increase in PAHs due to catechin addition was related to the free radicals generated by catechin. Figure 4B shows the free radical intensity of oil with different additions of catechin after heating at 160 °C for 20 min; the higher the peak, the more free radicals in the analytes. Therefore, it can be seen from the graph that the intensities of free radicals when catechin was added at 0.005% and 0.01% were higher than the control group, and with an increase in catechin addition, catechin showed an inhibitory effect on free radicals.

According to previous studies, phenolic-rich components have a better inhibitory effect on the formation of PAHs [35]. However, in this study, it was found that at certain concentrations, the addition of catechin not only increased the free radical contents in the samples, but also led to an increase in PAHs. Wang et al. found higher levels of both PAH8 and free radicals after margination of chicken wings with phenolic-rich beer than the control, and the free radical contents were positively correlated with the PAH8 contents [36]. Similar results were found in a study by Jongberg et al. [37]. This suggested that phenolic compounds may produce reactive oxygen species, such as hydrogen peroxide and hydroxyl radicals, through reductive coupling with oxygen at high temperatures, and caused oxidative damage to lipids as well as their own oxidation to phenoxy radicals, thus leading to a system more favorable to form PAHs.

From the results of free radical detection combined with PAH4 generation, the generation of PAH4 at high temperature in the more active stage of free radicals was positively correlated with free radicals, and the intensity of free radicals in the catechin-added samples also showed a correlation with the contents of PAH4.

### 3.6. FT-IR Spectroscopy Analysis

In order to more clearly investigate the role of catechin on the formation of PAHs during the heat treatment of *C. oleifera* oil, this study also used triglycerides as the object to study the liposome system and verified these results using FT-IR. The FT-IR spectra indicated specific functional groups and their infrared absorption wavelengths. Clearly visible variations based on the position and intensity of some bands were used to explain the structural changes of the triglycerides. 

**Figure 5:**
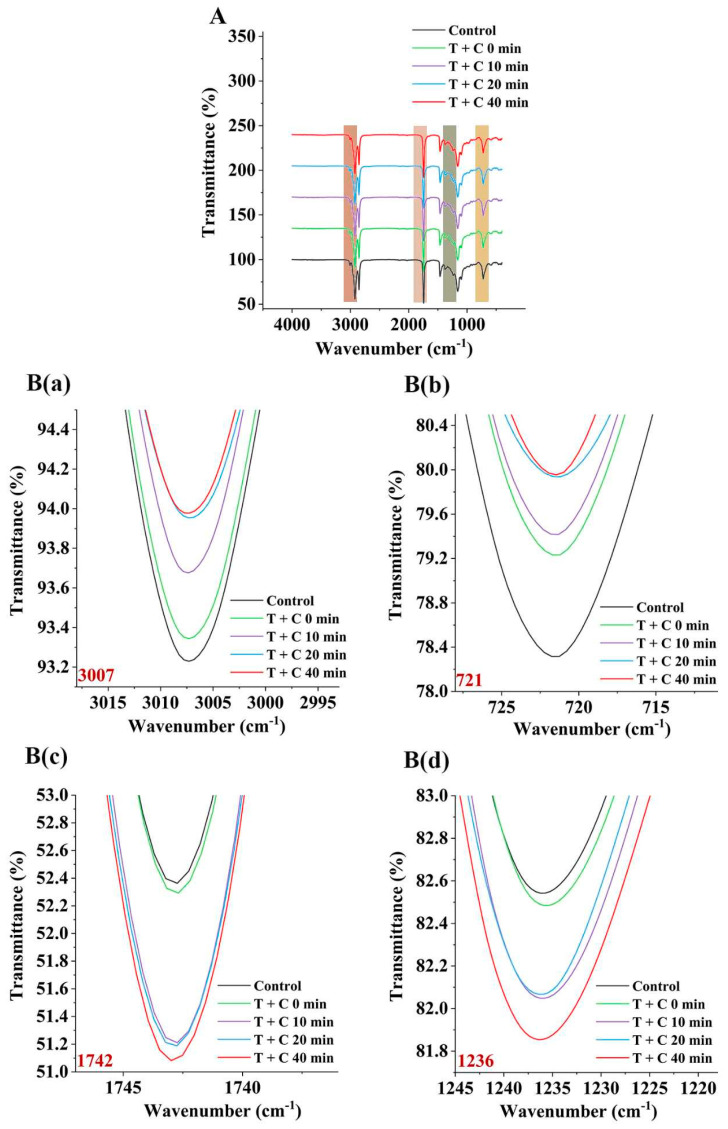
FT-IR spectra of triolein added with catechin heated for different times at 160 °C (**A**); Several characteristic peaks of (**A**): 3007 cm^−1^ (**B**) (**a**), 721 cm^−1^ (**B**) (**b**), 1742 cm^−1^ (**B**) (**c**), 1236 cm^−1^ (**B**) (**d**).

As shown in Figure 5A, the spectrum of triglyceride containing four major peaks near 3007, 1742, 1236, and 721 cm^−1^ showed the stretching vibrational positions of the unsaturated CH=CH, C=O stretching, C−O stretching, and CH=CH bending out of the plane, respectively; the two strong peaks near 2852 and 2921 cm^−1^ corresponded to the asymmetric stretching and symmetric stretching of −CH_2_, respectively; 1462 cm^−1^ corresponded to −CH_2_ bending; 1376 cm^−1^ corresponded to −CH_3_ bending; and 1119 and 1097 cm^−1^ both corresponded to C−O stretching [38]. In the figure, the higher the transmittance of the peak, the higher the contents of the specific functional groups. It was observed that the 2921 and 2852 cm^−1^ positions and transmittances of the samples, with and without catechin, were relatively stable after heat treatment because these two positions corresponded to the methyl and methylene groups, respectively, which are the most stable segments of triglycerides. It was also shown in several spectral bands studied that the transmittance of the peaks at 3007 and 721 cm^−1^ increased with heating time, and the spectra of the catechin-added samples showed higher transmittance than those of the non-catechin-added samples (Figure 5B). These results confirmed the degradation and oxidation of the lipids occurred during heating, more hydrolysis and oxidation reactions occurred in the functional groups of the oil samples, and the added catechin at high temperature that may be coupled with oxygen reduction to produce reactive oxygen species, such as hydrogen peroxide and hydroxyl radicals, causing oxidative damage to lipids. This result was consistent with the result of Section 3.3 in this study.

It is worth mentioning that the transmittances and peak positions at 1742 and 1236 cm^−1^ in the spectra of the oil samples after heat treatment also changed, with the peak at 1742 cm^−1^ reaching the lowest transmittance after 40 min of heating with the addition of catechin, followed by the samples at 10 min and 20 min, and the transmittance of the two samples were relatively close (Figure 5B). This indicates that the amount of stretching C=O vibrations of their ester-carbonyl chromophores increased slightly over time. This may have resulted from the breakdown of the acylglycerol ester bond and the oxidation of the C=C group to the C=O bond, causing the peak position shifted to 1744 cm^−1^. This change may be related to the overlap of the functional group of the aldehyde or other secondary oxidation products with the ester carbonyl functional group of the triglyceride. In the induction period of the first 20 min, aldehydes and other oxidation products were gradually generated, while some of them are transformed into other substances. In the oxidation period after 20 min, aldehydes and other oxidation products were generated in large quantities, so the transmittance continued to decrease. This result was consistent with the result of Section 3.4 in this study. Similar results were observed for the 1236 cm^−1^ bands, where the lowest transmittance was observed after the addition of catechin for 40 min, followed by the samples at 10 min and 20 min, and the peak positions were shifted to 1235 cm^−1^ (Figure 5B). However, these changes were due to the breakdown of C=C bonds and the formation of C-H units. Although the spectra in this experiment could not directly show the formation of PAHs, the FT-IR spectral results were consistent with the above chemical analysis results of the oxidative degradation of fatty acids, free radical changes, formation of PAHs intermediates, and degradation and oxidation of catechin, which further confirmed the influence of catechin on PAH formation. 

### 3.7. Analysis of Catechin in Thermal Processing

**Figure 6:**
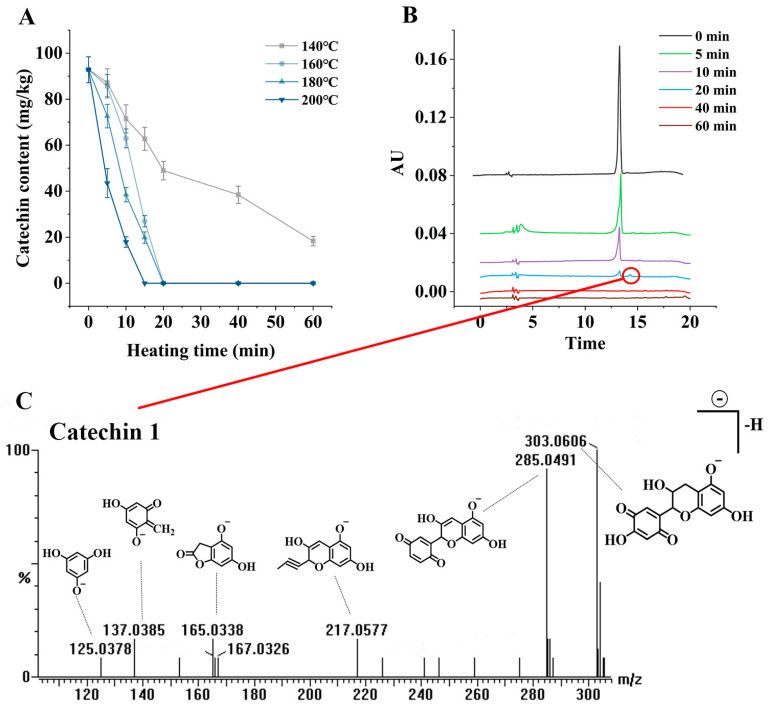
Contents of catechin at different temperatures with heating time (**A**); Chromatograms of catechin and catechin 1 at 160 °C for different heating times (**B**); Mass spectrum of catechin 1 and speculated fragment structure (**C**).

Figure 6A shows the changes in catechin contents during the heating of *C. oleifera* oil containing 0.01% catechin at different temperatures (140 °C, 160 °C, 180 °C, and 200 °C) for 60 min. As seen in the figure, the contents of catechin decreased significantly (*p* < 0.05) with time at all temperatures. At the high temperatures of 180 °C and 200 °C, the catechin contents decreased rapidly when heated for about 15 min, and completely degraded by 20 min, which may have been due to the volatilization of catechin and the loss of thermal reactions dominated in the high-temperature system, resulting in faster consumption of catechin. Similar results emerged in Liu’s study of TBHQ loss patterns in heating systems [39]. It was also found that the separately added catechin produced a new substance, named catechin 1, during the heating. Figure 6B shows the liquid chromatograms of 0.01% catechin when heated at 160 °C for 0, 5, 10, 20, 40, and 60 min. The retention time of catechin was 13.92 min, and the retention time of catechin 1 was 14.31 min. It can be seen that the peak represented by the catechin gradually decreased in the first 20 min, and a new peak appeared behind catechin at about 20 min. At 40 min, both catechin and catechin 1 were degraded and absent, thus, it was hypothesized that catechin 1 was the oxidation product of catechin in this system. The catechin 1 was then isolated and detected using UPLC-QTOF-MS (Figure 6C). According to the mass-to-charge ratio (*m/z*) and the previously reported tea polyphenols under the action of free radicals, the hydrogen of the phenolic hydroxyl group of the A ring itself is converted into phenoxy free radicals, and then further converted into quinones, so it was speculated that catechin 1 was a quinone converted from catechin after oxidation [40]. When all the catechin was degraded, the quinones disappeared quickly.

The overall results showed that throughout the thermal reaction, catechin participated in some chemical reactions to degrade themselves; moreover, by comparing the amount of PAH4 production and performing correlation analysis, it was found that the PAH4 contents continued to increase during the phase of gradual degradation of catechin, and the catechin contents showed a significant negative correlation with PAH4 production when the catechin addition was <0.02%. 

**Figure 7:**
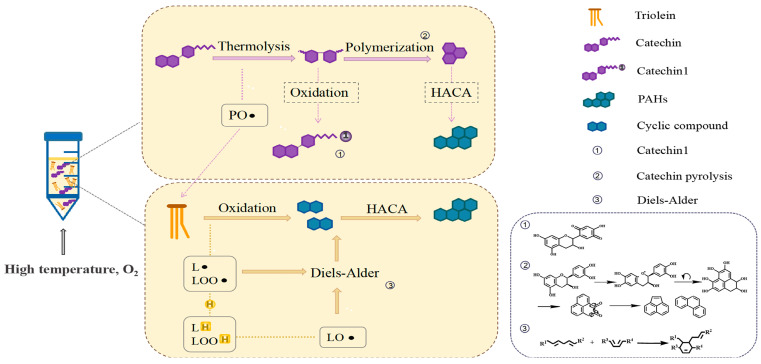
Speculative path of PAH4 formation in *C. oleifera* oil.

During the induction period of lipid oxidation, PAH4 were generated rapidly. Figure 7 shows the speculative path of PAH4 formation in *C. oleifera* oil. When catechin addition was <0.02%, more free radicals were produced than quenched, resulting in free radicals that attacked triglyceride and caused the oxidation and decomposition of fatty acids. Meanwhile, the hydrogenoxides produced may also generate cyclic compounds, such as cyclohexene through intramolecular cyclization, which eventually generated benzene rings. Molecular growth was then achieved through HACA (hydrogen atoms removed-acetylene molecules added) mechanism to form PAHs [41]. This led to an increase in PAHs intermediates and PAH4. Moreover, unstable groups such as C−O, C−OH, and C−C in the benzene ring side chain of catechin itself would break at high temperature, and the C=C bond on the benzene ring skeleton would break and polymerize to form naphthalene, anthracene, and other dense aromatic ring compounds [42], which eventually led to an increase in PAH4 in the system.

## 4. Conclusions

In summary, this study suggested that oxidative decomposition of catechin at high temperature may be involved in the formation of PAHs. The results showed that the rapid growth of PAH4 in crude oil was associated with phenolic compounds compared to refined oil. Therefore, catechin was selected as the subject after the experiment and the effect of catechin on PAH4 formation in *C. oleifera* oil under high temperature was investigated. It was found that the formation of PAHs was inhibited when the catechin addition was >0.02%, while it was involved in the formation of PAHs when the addition of catechin was <0.02%. We further found the oxidative cleavage reaction of catechin at high-temperature, which generated free radicals attacking triglycerides and promoted the formation of PAHs intermediates. As well as the cyclization reaction of catechin itself after cleavage, it led to the increase of PAH4 content in the system. Therefore, in order to control the formation of PAHs in oil rich in phenolic compounds, prolonged use of oil at high temperature (>140 °C) is not recommended. When the oil uses phenol as an antioxidant, the phenol, with its better heat resistance, should be selected in the process of high-temperature use, and the appropriate addition amount should be screened out.

## Figures and Tables

**Table 1:** Fatty acid composition and lipid companion contents of crude *C. Oleifera* oil and refined *C. Oleifera* oil during heating.

**Heating Time (h)**	**Fatty Acid (g/100 g)**											
	**CCO**						**RCO**					
	**C16:0**	**C18:0**		**C18:1**		**C18:2**	**C16:0**	**C18:0**		**C18:1**		**C18:2**
0	8.11 ± 0.08 ^d^	2.36 ± 0.08 ^a^		80.15 ± 0.11 ^a^		9.44 ± 0.06 ^a^	9.82 ± 0.08 ^c^	2.56 ± 0.06 ^a^		78.18 ± 0.07 ^a^		8.81 ± 0.11 ^a^
1	8.29 ± 0.08 ^c^	2.45 ± 0.04 ^a^		79.37 ± 0.09 ^b^		9.31 ± 0.08 ^ab^	9.89 ± 0.08 ^bc^	2.59 ± 0.04 ^a^		78.07 ± 0.05 ^ab^		8.71 ± 0.06 ^ab^
2	8.42 ± 0.06 ^bc^	2.57 ± 0.18 ^a^		78.84 ± 0.13 ^c^		9.16 ± 0.06 ^bc^	10.02 ± 0.06 ^abc^	2.67 ± 0.18 ^a^		77.79 ± 0.06 ^bc^		8.56 ± 0.06 ^bc^
3	8.47 ± 0.04 ^abc^	2.37 ± 0.08 ^a^		78.58 ± 0.06 ^d^		9.13 ± 0.06 ^c^	10.04 ± 0.14 ^ab^	2.70 ± 0.11 ^a^		77.73 ± 0.13 ^bc^		8.48 ± 0.13 ^bc^
4	8.45 ± 0.11 ^abc^	2.41 ± 0.06 ^a^		78.42 ± 0.06 ^de^		9.02 ± 0.05 ^cd^	10.10 ± 0.02 ^ab^	2.74 ± 0.12 ^a^		77.67 ± 0.13 ^c^		8.42 ± 0.09 ^c^
5	8.51 ± 0.14 ^ab^	2.36 ± 0.08 ^a^		78.31 ± 0.11 ^ef^		8.88 ± 0.08 ^d^	10.11 ± 0.08 ^ab^	2.71 ± 0.13 ^a^		77.56 ± 0.18 ^c^		8.38 ± 0.05 ^c^
6	8.61 ± 0.09 ^a^	2.53 ± 0.06 ^a^		78.19 ± 0.08 ^f^		8.60 ± 0.11 ^e^	10.14 ± 0.078 ^a^	2.78 ± 0.15 ^a^		77.44 ± 0.28 ^c^		8.35 ± 0.11 ^c^
**Heating Time (h)**	**Total Phenols Contents (mg/kg)**				**Phytosterol Contents (mg/g)**				**Tocopherol Contents (mg/kg)**			
	**CCO**		**RCO**		**CCO**		**RCO**		**CCO**		**RCO**	
0	36.92 ± 2.70 ^a^		17.20 ± 1.98 ^a^		3.87 ± 0.11 ^a^		3.28 ± 0.16 ^a^		249.09 ± 5.35 ^a^		ND	
1	27.65 ± 2.65 ^b^		14.42 ± 1.57 ^ab^		3.70 ± 0.28 ^a^		3.16 ± 0.29 ^a^		165.56 ± 4.01 ^b^		ND	
2	21.03 ± 2.76 ^c^		13.21 ± 2.24 ^ab^		3.51 ± 0.14 ^ab^		3.11 ± 0.19 ^a^		139.24 ± 3.27 ^c^		ND	
3	20.12 ± 2.65 ^cd^		12.13 ± 1.98 ^ab^		3.37 ± 0.22 ^b^		3.05 ± 0.14 ^a^		121.77 ± 5.55 ^d^		ND	
4	18.06 ± 2.09 ^d^		11.10 ± 2.60 ^b^		3.20 ± 0.78 ^bc^		3.00 ± 0.56 ^a^		80.52 ± 2.26 ^e^		ND	
5	15.42 ± 1.82 ^e^		10.16 ± 2.76 ^b^		3.04 ± 0.16 ^c^		2.94 ± 0.98 ^a^		53.44 ± 4.94 ^f^		ND	
6	14.89 ± 1.34 ^e^		9.97 ± 2.84 ^b^		3.00 ± 0.27 ^c^		2.93 ± 0.41 ^a^		43.58 ± 4.02 ^f^		ND	
Decreasing percentage (%)	59.7		42.0		22.5		10.7		82.5		0	

Note: ND—not detected. The value carrying different letters are significantly different (*p* < 0.05) from control and each other when comparing all pairs of columns. Results presented are the means of three values followed by their standard deviation. n = 3. Decreasing percentage (%) = (C_0_ − C_6_)/C_0._ C_0_: The contents of lipid companion when heating time was 0 h; C6: The contents of lipid companion when heating time was 6 h.

**Table 2:** Pearson correlation coefficients of heating time, fatty acid composition, lipid companion, and PAH4 in crude *C. Oleifera* oil.

	PAH4	Heating Time	C16:0	C18:0	C18:1	C18:2	Total Phenols	Phytosterol	Tocopherol
PAH4	1	-	-	-	-	-	-	-	-
Heating time	0.964 **	1	-	-	-	-	-	-	-
C16:0	0.974 **	0.943 *	1	-	-	-	-	-	-
C18:0	0.534	0.457	0.699	1	-	-	-	-	-
C18:1	−0.992 **	−0.930 *	−0.979 **	−0.606	1	-	-	-	-
C18:2	−0.827	−0.930 *	−0.866	−0.512	0.780	1	-	-	-
Total phenols	−0.982 **	−0.912 *	−0.983 **	−0.661	0.997 **	0.771	1	-	-
Phytosterol	−0.983 **	−0.993 **	−0.953 *	−0.466	0.959 **	0.885 *	0.942 *	1	-
Tocopherol	−0.998 **	−0.960 **	−0.977 **	−0.547	0.989 **	0.834	0.980 **	0.976 **	1

Note: * *p* < 0.05; ** *p* < 0.01.

**Table 3:** Fatty acid composition of *C. oleifera* oil added with catechin at different heating temperatures, heating times, and different addition of catechin.

T (°C)	t (min)	Catechin Addition (%)	Fatty Acid (g/100 g)			
			C16:0	C18:0	C18:1	C18:2
0	60	0.010	8.11 ± 0.08 ^fg^	2.36 ± 0.08 ^e^	80.15 ± 0.11 ^a^	9.44 ± 0.06 ^a^
140	60	0.010	9.11 ± 0.08 ^b^	2.53 ± 0.06 ^cde^	78.77 ± 0.05 ^b^	7.94 ± 0.29 ^c^
160	60	0.010	9.27 ± 0.06 ^ab^	2.77 ± 0.04 ^ab^	78.25 ± 0.16 ^e^	6.32 ± 0.27 ^d^
180	60	0.010	9.30 ± 0.03 ^a^	2.87 ± 0.01 ^a^	78.20 ± 0.09 ^e^	6.31 ± 0.23 ^d^
200	60	0.010	9.35 ± 0.06 ^a^	2.91 ± 0.04 ^a^	78.15 ± 0.11 ^e^	6.24 ± 0.21 ^d^
160	0	0.010	8.11 ± 0.08 ^fg^	2.36 ± 0.08 ^e^	80.15 ± 0.11 ^a^	9.41 ± 0.03 ^ab^
160	5	0.010	8.17 ± 0.06 ^efg^	2.40 ± 0.04 ^de^	78.78 ± 0.04 ^b^	9.36 ± 0.06 ^ab^
160	10	0.010	8.23 ± 0.06 ^defg^	2.41 ± 0.02 ^de^	78.61 ± 0.10 ^bcd^	9.28 ± 0.09 ^ab^
160	15	0.010	8.28 ± 0.09 ^def^	2.44 ± 0.02 ^de^	78.58 ± 0.13 ^bcd^	9.19 ± 0.06 ^ab^
160	20	0.010	8.32 ± 0.06 ^de^	2.57 ± 0.17 ^cd^	78.39 ± 0.19 ^cde^	8.94 ± 0.18 ^b^
160	40	0.010	8.87 ± 0.13 ^c^	2.63 ± 0.05 ^bc^	78.35 ± 0.13 ^de^	7.76 ± 0.37 ^c^
160	60	0.010	9.27 ± 0.06 ^ab^	2.77 ± 0.04 ^ab^	78.25 ± 0.16 ^e^	6.32 ± 0.27 ^d^
160	20	0.000	8.09 ± 0.08 ^g^	2.41 ± 0.04 ^de^	78.72 ± 0.09 ^b^	8.60 ± 0.23 ^a^
160	20	0.005	8.39 ± 0.06 ^d^	2.49 ± 0.04 ^cde^	78.37 ± 0.09 ^de^	8.31 ± 0.08 ^ab^
160	20	0.010	8.32 ± 0.06 ^de^	2.57 ± 0.18 ^cd^	78.39 ± 0.19 ^cde^	8.94 ± 0.19 ^b^
160	20	0.020	8.22 ± 0.04 ^defg^	2.37 ± 0.08 ^e^	78.67 ± 0.04 ^bc^	9.30 ± 0.02 ^ab^
160	20	0.040	8.30 ± 0.12 ^de^	2.41 ± 0.06 ^de^	78.81 ± 0.09 ^b^	9.37±0.16 ^ab^

Note: The value carrying different letters are significantly different (*p* < 0.05) from control and each other when comparing all pairs of columns. Results presented are the means of three values followed by their standard deviation (n = 3).

## Data Availability

Data is contained within the article or Supplementary Materials.

## Supplementary Materials

The following supporting information can be downloaded at: https://www.mdpi.com/article/10.3390/foods12050980/s1, The supplementary experimental method is the specific details of Section 2.4 related experiments.

## Abbreviations

AOCS: American Oil Chemists Society; AV: acid value; BaA: benzo(a)anthracene; BaP: benzo(a)pyrene; BbF: benzo(b)fluoranthene; CCO: crude *C. oleifera* oil; RCO: refined *C. oleifera* oil; Chr: chrysene; *C. oleifera*: *camellia oleifera*; HACA: hydrogen atoms removed-acetylene molecules added; IP: induction period; LOD, limit of detection; LOQ, limit of quantification; PAHs: polycyclic aromatic hydrocarbons; PAH4: sum of benzo(a)anthracene, benzo(b)fluoranthene, chrysene, benzo(a)pyrene; PAH8: sum of benzo(a)anthracene, chrysene, benzo(b)fluoranthene, ben-zo(k)fluoranthene, benzo(a)pyrene, dibenzo(a,h)anthracene, benzo(g,h,i)perylene, and indeno(1,2,3-c,d)pyrene; POV: peroxide value; FT-IR: Fourier Transform infrared spectroscopy; HPLC-FLD: high performance liquid chromatography-fluorescence detection.
