# Supplementary material for: Effect of Catechin on the Formation of Polycyclic Aromatic Hydrocarbons in Camellia oleifera Oil during Thermal Processing
Source: Foods. 2023 Feb 25;12(5):980. doi: 10.3390/foods12050980 (PMC10000653; doi:10.3390/foods12050980)
Supplement: Supplementary file 1 [file foods-12-00980-s001.zip › foods-2121657-supplementary.pdf]

2. Materials and Methods

2.4 Analysis of PAHs

Sample pretreatment: Weigh 0.50 g of oil to be cleaned. The samples were processed by MIP solid phase extraction method. SPE columns specially designed for MIP−PAHs were inserted into CNW12−position solid phase extraction vacuum unit for activation. The SPE column was activated by adding 5 mL ethyl acetate and 5 mL petroleum ether successively. At the end of activation, 2 mL samples were added to the column, and then washed with 3 mL n−hexane in a 10 mL centrifuge tube. The loading rate was controlled to be less than 1 mL/min. After sample loading, 5 mL petroleum ether was added to the SPE column for leaching to further wash away impurities. After leaching, 10 mL ethyl acetate was added for elution. The eluent was collected in a 10 mL centrifuge tube and dried with nitrogen. The eluent was dissolved with acetonitrile for 1 min by ultrasound and volume was fixed to 1 mL. Pass 0.22 μm filter membrane for sampling.

Chromatographic conditions: LC−PAH column (250 mm×4.6 mm×5 μm); Sample size: 20 μL; Column temperature: 25 ºC; Mobile phase: A water, B acetonitrile; Gradient elution procedure: 0~5 min, 50% A; 5~30 min, 50% A uniformly increased to 100% A: 30~45 min, 100% A. The detector was a fluorescence detector with excitation wavelength of 260 nm and emission wavelength of 420 nm.

The linear range of the method was 1−100 μg/L. The correlation coefficient was greater than 0.999. The limits of detection (LOD) of Chr, BaA, BbF, and BaP were 0.25, 0.29, 0.023, and 0.061 μg/kg. Meanwhile, the corresponding limits of quantitation (LOQ) were 0.85, 0.98, 0.076, and 0.21 μg/kg, respectively. The recovery rate of PAH4 ranged from 70.2% to 110%.

Calculation of LOD and LOQ: An aliquot of 0.5 g oil sample was weighed and PAHs mixed standard solution was added quantitatively to achieve concentrations of 2 μg/kg, 10 μg/kg, and 20 μg/kg, respectively, for 6 repetitions. The detection limit of the object to be measured is set according to international regulations. The concentration under S/N=3 is defined as the detection limit (LOD), while the concentration under S/N=10 is defined as the limit of quantitation (LOQ).

Method accuracy and precision: An aliquot of 0.5 g oil sample was weighed, an appropriate amount of PAHs standard solution was added, pre−treatment was carried out according to Method 2.3, and an addition recovery experiment was conducted. Six parallel experiments were conducted at each addition level to calculate precision and recovery rate. The calculation formula was:

$$\text{Recovery rate (\%)} = \frac{Xs - X0}{M} * 100\%$$

Xs: Actual measured amount of PAHs in the samples added with PAHs,

X_0: Actual measured amount of PAHs in the blank sample,

M: Amount of PAHs added.

2.5 Determination of physicochemical parameters, fatty acid composition, minor constituents content, and heating products

physicochemical parameters: Acid value and Peroxide value of this were determined based on the AOCS official methods (AOCS, 2009).

Fatty acid composition: About 0.2 mg of walnut oil was dissolved in 2.0 mL n−hexane and

added 0.5 mL 2 mol L^{-1} KOH–CH_3OH to prepare fatty acid methyl esters as described in our earlier study. The fatty acid was analyzed using a 7820A gas chromatograph (GC) (Agilent, Palo Alto, CA, USA) and a Trace TR−FAME capillary column (0.25 μm, 60 m × 0.25 mm, Thermo Fisher, Waltham, MA, USA). Operating conditions were as follows: Nitrogen as carrier gas with linear velocity of 1.0 mL min^{-1}, flame ionization detector (FID) (Thermo Fisher) and injector temperature at 250°C, split ratio 1:100, injection volume 1.0 μL, column was held at 60°C for 3 min, and then programmed at 5°C $min^{-1}$to 170°C and hold for 15 min and finally increased to 220°C at 2°C min^{-1} and held for 10 min.

Minor constituents content:

α−tocopherols: Oil of 0.50 g was weighted and diluted with n−hexane in 10 mL volumetric flask and determined by high−performance liquid chromatographic (LC−20AT, Shimadzu, Tokyo, Japan). The ultraviolet detector was performed on SPD−20A (Shimadzu) installed with a silica column (5 μm, 4.6 × 250 mm, Hanbon, Jiangsu, China). The injection volume of sample was 20 μL, column temperature was 30°C, and the mobile phase was hexane/isopropanol (98.5/1.5, v/v) with a rate of 1.0 mL/min. The determining wavelength set at 295 nm was used. Through the comparison of standards, α−, β−, γ−, and δ−tocopherols were identified and quantified, and their contents were mg/kg.

Phytosterols: Phytosterols analyses were analyzed by a gas chromatograph−mass spectrum system (Thermo Fisher, USA) equipped with a FID as described. First, 100 mg of the fat was mixed with 0.2 mL of 0.825 mg/mL 5 α−cholestane and 2 mL of 2 mol/L KOH–CH_3CH_2OH. The mixture was heated at 85°C for 1 h and then cooled, and 5 mL of hexane and 2 mL of distilled water were added to extract the unsaponifiables three times. The product was then

dried by nitrogen and silylated with 400 μL BSTFA + TMCS at 70°C for 30 min, and finally was dissolved by 1 mL hexane. Next, 1 μL of the prepared sample was injected into the gas chromatograph and separated by a DB−5 capillary column (0.25 μm, 30 m × 0.25 mm, Agilent, USA). The initial column temperature was set at 200°C for 1.0 min, then increased to 300°C at a rate of 10°C/min and maintained for 18 min. Both of the FID and injector temperatures were 290°C. Carrier gas (helium) was 1 mL/min and the split ratio was 1:100. In addition, the ion source and transmission line temperature were 280 and 250°C, respectively. The ionization mode was EI and the mass range (m/z) was 50–500. Phytosterols contents were reported in mg/kg.

Total phenols: pretreatment: Solid phase extraction (DIOL−SPE) column was used to extract polyphenols from oil. First, 6 mL methanol and 6 mL hexane were used to activate the SPE column. The camellia seed oil sample was weighed 1.5 g, accurate to 0.001 g, dissolved in 6 mL hexane through the column, and then cleaned the column twice with 3 mL hexane. Then, 4 mL hexane−ethyl acetate (v : v=90 : 10) was added through the column. Finally, methanol solution was added to the elute and collected in a 10 mL volumetric flask. After constant volume, 5 mL was removed and placed in another 10 mL volumetric flask, 0.5 mL folinol was added for 3 min, and 1 mL of 10% Na_2CO_3 solution was added. After constant volume, it was left in the dark for 2 h. The absorbance was measured by spectrophotometer at 765nm wavelength.

PAHs intermediates: For analysis, 3 ± 0.02 g oil samples and 20 μL of standard solution (1,2−dichlorobenzene, 1031 μg/mL) were added to a 20 mL vial. A 50 μm/30 μm DVB/CAR/PDMS fiber (Supelco Inc., Bellefonte, PA) was used for headspace sampling.

Volatile compounds were analyzed by GC–MS using a TSQ Quantum XLS (Thermo, USA) operating in electron ionization mode (EI, 70 eV). After 30 min of extraction at 60°C, the fiber was immediately desorbed into the GC–MS injection port at 250°C for 5 min. Volatile compounds were analyzed on a GC–MS apparatus using a TSQ Quantum XLS (Thermo Fisher Scientific, San Jose, CA, USA) operating in electron ionization mode (EI, 70 eV). The DB−WAX MS column (30 m × 0.25 mm × 0.25 μm) was programmed from 70°C (with holding for 3 min) to 130°C at 5°C min^{-1} and then rose to 220°C at 8°C min^{-1} (with holding for 10 min). Finally, the temperature rose to 250°C at 10 °C min^{-1} (hold for 5 min). Helium was used as carrier gas (1.0 mL min^{-1}).
